# Comparison of the oral microbiome in mouthwash and whole saliva samples

**DOI:** 10.1371/journal.pone.0194729

**Published:** 2018-04-11

**Authors:** Xiaozhou Fan, Brandilyn A. Peters, Deborah Min, Jiyoung Ahn, Richard B. Hayes

**Affiliations:** 1 Department of Population Health, NYU School of Medicine, New York, New York, United States of America; 2 NYU Laura and Isaac Perlmutter Cancer Institute, New York, New York, United States of America; Babasaheb Bhimrao Ambedkar University, INDIA

## Abstract

Population-based epidemiologic studies can provide important insight regarding the role of the microbiome in human health and disease. Buccal cells samples using commercial mouthwash have been obtained in large prospective cohorts for the purpose of studying human genomic DNA. We aimed to better understand if these mouthwash samples are also a valid resource for the study of the oral microbiome. We collected one saliva sample and one Scope mouthwash sample from 10 healthy subjects. Bacterial 16S rRNA genes from both types of samples were amplified, sequenced, and assigned to bacterial taxa. We comprehensively compared these paired samples for bacterial community composition and individual taxonomic abundance. We found that mouthwash samples yielded similar amount of bacterial DNA as saliva samples (*p* from Student’s t-test for paired samples = 0.92). Additionally, the paired samples had similar within sample diversity (*p* from = 0.33 for richness, and *p* = 0.51 for Shannon index), and clustered as pairs for diversity when analyzed by unsupervised hierarchical cluster analysis. No significant difference was found in the paired samples with respect to the taxonomic abundance of major bacterial phyla, *Bacteroidetes*, *Firmicutes*, *Proteobacteria*, *Fusobacteria*, and *Actinobacteria* (FDR adjusted q values from Wilcoxin signed-rank test = 0.15, 0.15, 0.87, 1.00 and 0.15, respectively), and all identified genera, including genus *Streptococcus* (q = 0.21), *Prevotella* (q = 0.25), *Neisseria* (q = 0.37), *Veillonella* (q = 0.73), *Fusobacterium* (q = 0.19), and *Porphyromonas* (q = 0.60). These results show that mouthwash samples perform similarly to saliva samples for analysis of the oral microbiome. Mouthwash samples collected originally for analysis of human DNA are also a resource suitable for human microbiome research.

## Background

Emerging evidence shows that oral microbiota is closely tied to oral diseases, including periodontitis and dental caries [1], and potentially to systemic diseases, including diabetes [2], cardiovascular disease [3], and several types of cancer [4–7]. While it is a commonplace that good oral health is related to good systemic health [8], only recently has it become possible to investigate the underlying microbial basis of this association. Two advances are noteworthy in this regard. Fostered by the Human Microbiome Project [9], laboratory techniques are now available to efficiently characterize the full microbiome complement of biologic samples through next-generation sequencing technology and associated bioinformatic tools [10, 11]. Secondly, large collections of oral wash samples containing human and microbial DNA have been collected in epidemiologic cohort studies and stored for research on the future development of disease.

Several large-scale epidemiologic collections of oral wash samples, each involving more than 50,000 subjects [12–14], have been carried out using Scope (Procter & Gamble, Cincinnati, OH), a commercially available mouthwash, however, there is a need to determine whether the use of this product, for ease of sample collection, influenced microbiome composition, as compared to simple collection of saliva. We assessed the oral bacterial profiles from next-generation sequencing of the 16S rRNA gene in samples collected using Scope mouthwash as compared to simple saliva collection from 10 healthy subjects. We hypothesize that the bacterial profiles in these two types of oral samples collected from the same individuals are similar in composition. Comprehensive comparisons in these paired samples were conducted with respect to community composition and specific taxonomic abundance.

## Methods

### Sample collection

This study was carried out in strict accordance with the recommendations with The Code of Ethics of the World Medical Association (Declaration of Helsinki) for experiments involving humans. All participants provided informed consent and all protocols were approved by the New York University School of Medicine Institutional Review Board (Permit Number: S12-00721). Four males and six females were enrolled at Department of Population Health, New York University Medical Center (S1 Table) with mean age 33.5 ± 13.2 years (range 25–70). All subjects signed informed consent and had not used antibiotics in the past 3 months. Before collection, subjects refrained from drinking and eating for at least 2 hours. Five mL saliva was collected by allowing saliva to accumulate on the floor of the mouth followed by expectoration into a specimen tube every 60 seconds [15]. After saliva sample collection, subjects were asked to swish vigorously with 10 mL Scope mouthwash with a 15 wt% alcohol content (Procter & Gamble, Cincinnati, OH) for 30 seconds, and then to expectorate into another specimen tube. Both saliva and mouthwash samples were transported to our laboratory and stored at -80°C within 10 minutes after collection.

### DNA extraction and 16S rRNA gene sequencing

Bacterial genomic DNA was extracted from 10 mouthwash, 10 saliva, and 2 blank mouthwash samples, using the MoBio PowerSoil DNA Isolation Kit (Carlsbad, CA), with the bead-beating method in the MoBio Powerlyzer instrument. We followed the manufacturer’s protocol, except adding a 65 °C heating step after the addition of Solution C1 of the MoBio PowerSoil DNA Isolation Kit. Illumina MiSeq16S rRNA gene sequencing was performed for the extracted DNA. Briefly, PCR amplicon libraries targeting the 16S rRNA encoding gene were produced using a barcoded primer set adapted for the Illumina HiSeq2000 and MiSeq [16]. DNA sequence data was then generated using Illumina paired-end sequencing. The fourth hypervariable (V4) region of the 16S rRNA gene (515F-806R), the most sensitive region as a marker for bacterial and phylogenetic analysis, was PCR amplified with region-specific primers and sequencer adapter sequences used in the Illumina flowcell [16–18]. Each 25 μL PCR reaction contained 9.5 μL of MO BIO PCR Water (Certified DNA-Free), 12.5 μL of QuantaBio’s AccuStart II PCR ToughMix (2x concentration, 1x final), 1 μL Golay barcode tagged Forward Primer (5 μM concentration, 200 pM final), 1 μL Reverse Primer (5 μM concentration, 200 pM final), and 1 μL of template DNA. Five ng genomic DNA was used as the template in 25 uL PCR reaction buffer for 16S rRNA amplicon preparation. The conditions for PCR were as follows: 94 °C for 3 minutes to denature the DNA, with 35 cycles at 94 °C for 45 s, 50 °C for 60 s, and 72 °C for 90 s; with a final extension of 10 min at 72 °C to ensure complete amplification. Amplicons were then quantified using PicoGreen (Invitrogen) and a plate reader (Infinite^®^ 200 PRO, Tecan). Once quantified, volumes of each of the products was pooled into a single tube so that each amplicon is represented in equimolar amounts. The pools were then cleaned using AMPure XP Beads (Beckman Coulter), and quantified by fluorometer (Qubit, Invitrogen). After quantification, the molarity of the pool was determined and diluted to 2 nM, denatured, and then diluted to a final concentration of 6.75 pM with a 10% PhiX spike for sequencing on the Illumina MiSeq. Amplicons were sequenced on a 151bp x 12bp x 151bp MiSeq run using customized sequencing primers and procedures [16].

### Statistical analysis

The Illumina-sequenced amplicon data was processed by using the DADA2 pipeline for quality filtering and construction of the operational taxonomic units (OTUs) [19]. DADA2 implements a novel algorithm that models the errors introduced during amplicon sequencing, and uses that error component to infer the true sample composition. The filtered output sequences were assigned to taxonomy using the GreenGenes 13.8 release clustered at 97% identity as reference database (assignTaxonomy function, dada2 package, R Foundation) [19]. We further filtered the OTU table by removing environmental contaminants that presented in the blank samples.

α-diversity was assessed for the number of observed OTUs (richness) and the Shannon index (evenness). These α-diversity indices were calculated in 500 iterations of rarefied OTU tables with a minimum sequencing depth of 38,400 among all study subjects. The average over the iterations was taken for each participant. Student’s t-test for paired samples was used in the comparisons for α-diversity indices. β-diversity was assessed using Jensen-Shannon divergence (JSD) distance matrix. To examine the bacterial community profiles in paired samples, we used “pvclust” *R* package to perform hierarchical cluster analysis via function “hclust” and calculate the probability value (approximately unbiased [AU] *p*-value) for each cluster using bootstrap analysis [20]. AU *p*-value of a cluster is the frequency that it appears in the multistep-multiscale bootstrap replicates. The identified OTUs were classified into 17 phyla, 30 classes, 51 orders, 87 families, 145 genera, and 183 species according to their alignment with the GreenGenes reference database. Sequences reads were normalized by using the centered log-ratio transformation (clr) [21], with a uniform prior of 0.0001 added to each count value before transformation. Wilcoxon signed-rank tests were used to examine the relative abundance differences in paired samples. To account for multiple comparisons at each taxonomic level, we considered an FDR-adjusted *p*-value (q value) less than 0.10 as significant. All statistical tests were two-sided, and all statistical analyses were carried out using R version 3.4.0.

## Results

**Fig 1:**
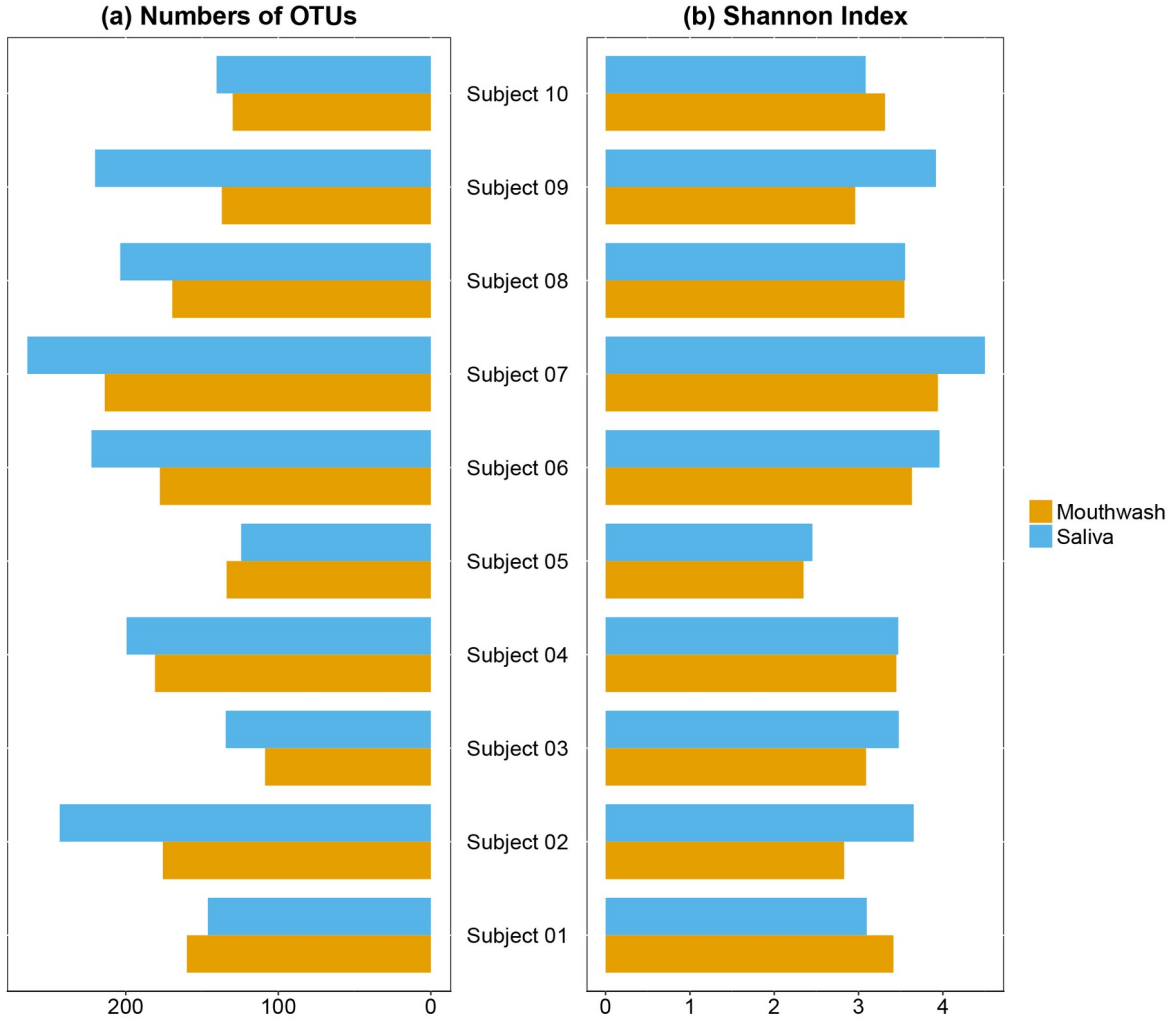
Alpha-diversity of oral bacterial communities in the paired mouthwash-saliva samples. Bar plots of number of observed OTUs (a) and Shannon Index (b) in paired mouthwash-saliva samples in 10 subjects. These indices were calculated for 500 iterations of rarefied OTU table with minimum sequencing depth of 38,400 among all study subjects, with the average over the iterations taken for each participant. No differences were found between mouthwash and saliva samples in α-diversity (*p* from paired t-test = 0.33 for richness, and 0.51 for Shannon index).

We obtained 2,488,884 sequence reads for analysis; mean values were 123,945 and 125,134 for mouthwash and saliva samples, respectively (paired t-test, *p* = 0.92). The within (α-) and between (β-) samples diversity were then used to examine the overall structure of the oral microbiota. For α-diversity, no difference were found in richness or evenness, measured by number of observed OTUs and the Shannon index in paired samples (Fig 1a and 1b, *p* = 0.33 and 0.51 for richness and Shannon index, respectively).

**Fig 2:**
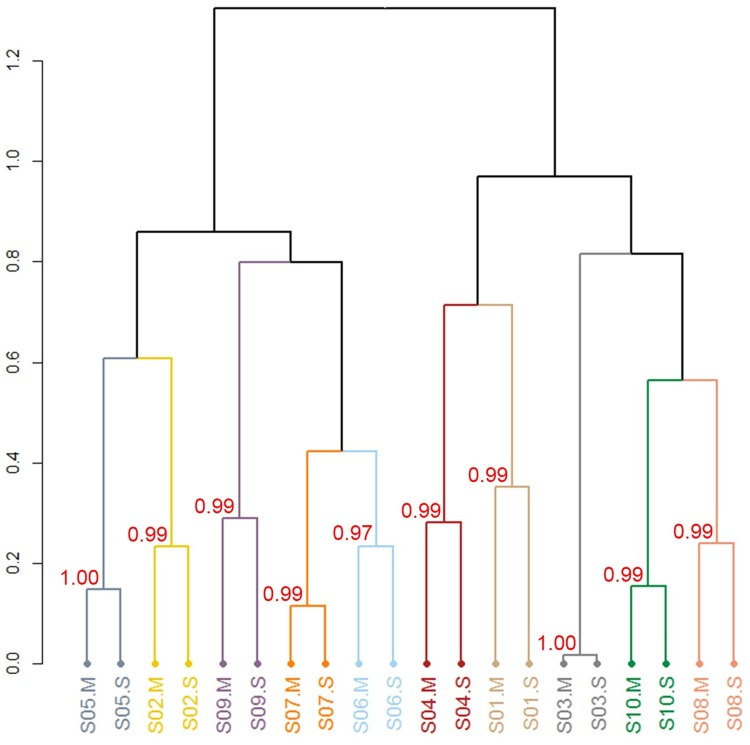
Beta-diversity of oral bacterial communities in the paired mouthwash-saliva samples. Hierarchical cluster analysis using JSD distance. AU (approximately unbiased) *p*-values, the unbiased bootstrap probability, ranged from 0.97 to 1.00 for all paired samples in hierarchical cluster analysis with number of 1,000 bootstrap replications. Cluster with AU ≥ 0.95 are considered to be strongly supported by data. S01-S10 indicate study subject 1 to 10. “M” indicates mouthwash sample and “S” indicates salivary sample.

In the analysis of β-diversity, the unsupervised hierarchical cluster analysis based on JSD distance matrix showed that the mouthwash and saliva sample from the same individual clustered exclusively with each other (Fig 2). AU *p*-values were 0.97 to 1.00 for all paired samples, using 1,000 bootstrap replications, which indicate the individual pairs were strongly supported by the data (Fig 2).

**Fig 3:**
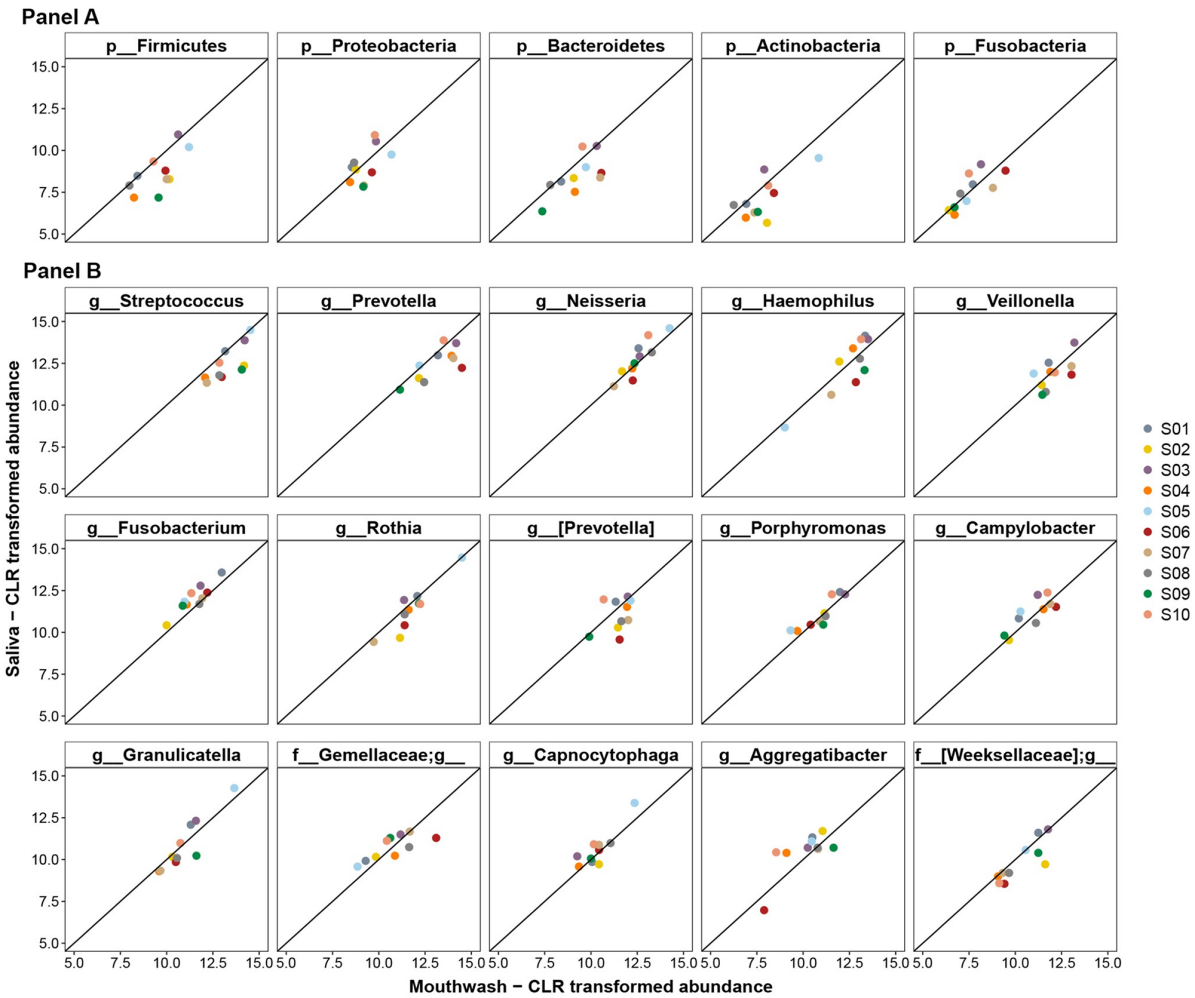
Correlation of the centered Log-Ratio (clr) transformed count of major bacteria phyla and genera in the paired mouthwash-saliva samples. Correlation of clr-transformed counts in mouthwash and saliva samples of major bacterial phyla (Panel A) and genera (Panel B). The x-axis represents the transformed counts in mouthwash samples, and the y-axis represents transformed counts in saliva samples. The straight line is the line of equality. All FDR adjusted q values from Wilcoxon signed-rank test for the comparison of the taxonomic abundance in paired samples were >0.05.

We next compared the taxonomic abundance for the paired samples (S2–S4 Tables). The 5 major bacterial phyla *Bacteroidetes*, *Firmicutes*, *Proteobacteria*, *Fusobacteria*, and *Actinobacteria* [22] account for 93.7–99.9% of the sequence reads in each sample. Their abundance showed high agreement in the paired saliva and mouthwash samples (Fig 3). No statistical differences were noted for the 5 phyla examined (S2 Table, FDR-adjusted q from Wilcoxin signed-rank test = 0.15, 0.15, 0.87, 1.00, 0.15, respectively). In Fig 3 we also highlighted fifteen “core” [23] genera that were present in all collected samples, which account for 83.1–95.0% of the sequence reads in each sample. None of these genera differed in the paired samples after FDR adjustment, including *Stretococcus* (q = 0.21), *Prevotella* (q = 0.25), *Neisseria* (q = 0.37), *Veillonella* (q = 0.73), *Fusobacterium* (q = 0.19), and *Porphyromonas* (q = 0.60), as well as the remaining genera shown in S3 Table. We also assessed differentials for 50 named species and found that none of their abundances differed in the paired samples, except for *Rothia aeria* (q = 0.098, S4 Table).

## Discussion

In this study, we found that the overall bacterial community composition was not significantly different between samples collected using mouthwash compared to simple saliva collection, consistent with a previous study [24]. We also demonstrated that the taxonomic abundance of the major bacteria was not different between paired mouthwash-saliva samples from the same individual. Thus, use of mouthwash to facilitate sample collection did not substantively alter the microbial profiles studied.

The main active ingredient in Scope mouthwash, cetylpyridinium chloride (CPC), has a broad antimicrobial spectrum with a rapid antiseptic effect on bacteria [25]. CPC non-selectively kills bacteria [26, 27] by altering the bacterial membrane function, which results in leakage of cytoplasmic material and ultimately the collapse of the intra-cellular equilibrium [27, 28]. Other ingredients, such as alcohol and essential oil, are used to increase solvability and help to penetrate plaque [29, 30], rather than to act as antibiotics [26]. Due to the rapid cessation of bacterial growth, the expectorated mouthwash preserves the undestroyed genomic information of bacteria, and it is unlikely that CPC mouthwash alters the composition of bacterial genomic information or its capacity for PCR amplification, as demonstrated by comparable sequencing depth in the paired samples in our study. Thus, the observed differences in low-abundance taxa, such as *Rothia aeria*, in the paired samples might be the result of within subject variability in repeated sampling, even if only minutes apart. Consistent with comparable amplification of sequence reads, our study also showed that the oral microbiome compositions are similar in paired samples using two different collection methods, with respect to overall bacterial community structure and abundance of the major taxa contributing to that structure, including *Streptococcus*, *Haemophilus*, *Neisseria*, *Prevotella*, *Veillonella*, and *Rothia*, and their constituent species, such as *S*. *anginosus*, *N*. *oralis*, and *R*. *mucilaginosa*.

Although we demonstrated comparability of the microbiome using these two collection procedures with freezing within 10 minutes of collection, we did not evaluate the impact of delayed time to freezer storage of samples, as may be the case in field epidemiologic studies. Here again, however, we expect that the bacteriostatic properties of the mouthwash would be of advantage in preventing bacterial replication between the time of collection and freezing.

In conclusion, we found similar oral bacterial profiles in paired saliva and mouthwash samples in 10 healthy subjects. The results indicate that the two collection methods have similar characteristics with respect to oral microbiome characterization and that frozen mouthwash samples are suitable for oral bacterial microbiome analysis.

## Supporting information

S1 Table Age and gender of ten study participants. (XLSX)

S2 Table Centered Log-Ratio transformed abundance of bacteria phyla in the paired mouthwash-saliva samples. (XLSX)

S3 Table Centered Log-Ratio transformed abundance of bacteria genera in the paired mouthwash-saliva samples. (XLSX)

S4 Table Centered Log-Ratio transformed abundance of previously named bacteria species in the paired mouthwash-saliva samples. (XLSX)
